# Toward the diagnosis of rare childhood genetic diseases: what do parents value most?

**DOI:** 10.1038/s41431-021-00882-1

**Published:** 2021-04-26

**Authors:** Samantha Pollard, Deirdre Weymann, Jessica Dunne, Fatemeh Mayanloo, John Buckell, James Buchanan, Sarah Wordsworth, Jan M. Friedman, Sylvia Stockler-Ipsiroglu, Nick Dragojlovic, Alison M. Elliott, Mark Harrison, Larry D. Lynd, Dean A. Regier

**Affiliations:** 1 Canadian Centre for Applied Research in Cancer Control, BC Cancer, Vancouver, Canada; 2 Nuffield Department of Population Health, Health Economics Research Centre, University of Oxford, Oxford, UK; 3 Department of Medical Genetics, University of British Columbia, Vancouver, Canada; 4 BC Children’s Hospital Research Institute, Vancouver, Canada; 5 Department of Pediatrics, Faculty of Medicine, University of British Columbia, Vancouver, Canada; 6 Division of Biochemical Genetics, BC Children’s Hospital, Vancouver, Canada; 7 Collaboration for Outcomes Research and Evaluation, Faculty of Pharmaceutical Sciences, University of British Columbia, Vancouver, Canada; 8 Centre for Health Evaluation and Outcomes Sciences, Providence Health Research Institute, Vancouver, Canada; 9 School of Population and Public Health, University of British Columbia, Vancouver, Canada

**Keywords:** Genetic testing, Economics, Next-generation sequencing

## Abstract

Genomic testing is becoming routine for diagnosing rare childhood genetic disease. Evidence underlying sustainable implementation is limited, focusing on short-term endpoints such as diagnostic yield, unable to fully characterize patient and family valued outcomes. Although genomic testing is becoming widely available, evidentiary and outcomes uncertainty persist as key challenges for implementation. We examine whether the current evidence base reflects public tolerance for uncertainty for genomics to diagnose rare childhood genetic disease. We conducted focus groups with general population parents in Vancouver, Canada, and Oxford, United Kingdom, to discuss expectations and concerns related to genomic testing to diagnose rare childhood genetic disease. Applying a purposive sampling technique, recruitment continued until thematic saturation was reached. Transcripts were analysed using thematic analysis. Thirty-three parents participated across four focus groups. Participants valued causal diagnoses alongside management strategies to improve patient health and wellbeing. Further, participants valued expanding the evidence base to reduce evidentiary uncertainty while ensuring security of information. Willingness to pay out of pocket for testing reflected perceived familial health benefit. Diagnostic yield fails to fully capture valued outcomes, and efforts to resolve uncertainty better reflect public priorities. Evaluations of genomic testing that fully integrate valued endpoints are necessary to ensure consistency with best practices and public willingness to accept the uncertain familial benefit.

## Introduction

Rare diseases are often complex, and can adversely impact physical and cognitive development, patient quality of life, and survival [1]. While each rare disease affects fewer than five in 10,000 people, over 9000 rare diseases have been described that, when combined, affect up to one in 16 people [2]. Predominantly manifesting in children, rare diseases include conditions such as idiopathic intellectual disability and early onset epilepsy. Approximately 39% of rare diseases have a suspected genetic origin [2]. Owing to disease heterogeneity and unknown variant pathogenicity, about half of patients with rare genetic diseases never receive a causal diagnosis [3, 4]. In addition to patient and family burden, a recent Canadian study found that healthcare systems spend up to $5600 per patient undergoing diagnostic testing and that parents accrue nearly $2000 in annual out-of-pocket costs [5]. Patients and families can undergo an extensive diagnostic odyssey without realizing any effective disease management or treatment strategies, with or without a genetic diagnosis [5–8].

Genomic technologies, such as next generation sequencing, enable faster diagnosis through simultaneous interrogation of a patient’s complete set of DNA (genome), protein-coding regions (exome) or multi-gene panels [7, 9]. Adoption of genomic testing as a clinical standard of care requires evidence of clinical benefit and cost-effectiveness, the latter typically established through comparing incremental costs with quality-adjusted life years (QALYs) gained [10, 11]. Quality-adjusted life years examine the impact of a health intervention by combining the preference-based value of a health state with time spent in that state, into a single index [12]. Epidemiological and clinical variables such as low population prevalence, variable phenotypic and patient heterogeneity, poorly established pathogenicity (disease causation), and a lack of effective genomics-informed treatments limit the ability to estimate patient impact attributable to genomic testing [13]. Clinical studies evaluating the validity of sequencing technologies are hindered by small sample sizes, and corresponding statistical imprecision, commonly using diagnostic yield as the primary endpoint [7, 14].

Economic evaluations similarly focus on diagnostic yield as a primary effectiveness outcome measure due to the unavailability of other kinds of evidence [15]. This emphasis on diagnosis as a primary outcome reflects the inability of instruments facilitating QALY calculations to incorporate the value of a genomic test on outcomes other than the ability to alter disease management, a concept termed personal utility [16, 17]. Personal utility––the benefits that patients ascribe to the spectrum of information derived from genomic technologies, regardless of their potential to improve health––is a key concept in childhood rare disease and has been extensively examined [1, 18–21]. Individual personal utility derived from genomic interventions may be a function of the need for repeat genomic testing or analysis, access to patient support, and return of uncertain or secondary findings [18, 19]. Incorporating personal utility beyond QALY estimates will allow health systems to make evidence- and value-based decisions about the adoption of genomic testing.

Despite a limited economic evidence base, jurisdictions around the world are increasingly reimbursing genomic technologies for rare diseases [22–26]. In the United Kingdom (UK), whole genome sequencing for suspected rare diseases is included for some diseases as part of the Genomic Medicine Service within the National Health Service [24]. Exome sequencing is similarly eligible or under consideration for provincial payment or reimbursement in select Canadian provinces [27].

An emerging pattern of reimbursement informed by limited evidence suggests public, decision maker, and clinician’s greater tolerance for uncertainty related to expected patient benefit [28–30]. Uncertain outcomes are driven by variation in the probability of a diagnosis being returned, known mutation pathogenicity, targeted treatment availability and access, as well as poorly established evidence available to estimate the direction and magnitude of treatment effect. Despite a considerable paucity of evidence to inform downstream patient impact alongside reimbursement, public willingness to consider value trade-offs under explicit conditions of unknown benefit is less explored. Published research either ignores personal utility when focusing on uncertainty in treatment effectiveness or emphasizes personal utility but ignores outcome uncertainty [31, 32]. Within publicly funded healthcare systems, capturing public values is recommended to ensure resource allocation decisions reflect priorities of those who will ultimately fund reimbursed interventions [32]. Private-payer systems such as the United States have also begun to initiate more patient-centered approaches to approval processes [33, 34]. The value that the public ascribes to uncertainty in quality of life gains and personal utility, is not well-established in the context of diagnosing rare childhood diseases.

### Objective and context

This qualitative work was conducted as part of a cross-jurisdictional economic evaluation of genomic sequencing to diagnose rare childhood genetic diseases, comparing two publicly funded health systems (Canada and the United Kingdom). This work will support the appropriate and sustainable adoption of genomic testing (whole genome and whole exome sequencing) to diagnose and guide treatment for children with rare diseases. Here we present focus groups with parents of unaffected children, across the two jurisdictions. The purpose of the qualitative work is to determine valued endpoints when considering reimbursement of genomics to diagnose rare childhood illness. Focus group findings will guide the development of a discrete choice experiment (DCE) that will be administered to members of the public in both Canada and the UK [35–37]. This approach allows for jurisdiction-specific preference-based utility estimates for the implementation of genomics to diagnose children with rare diseases [38]. Reporting of this work adheres to the consolidated criteria for reporting qualitative studies (COREQ) guidelines [39].

## Materials and methods

### Instrument development

The focus group topic guide was developed following the completion of a scoping review of preference elicitation studies for rare childhood diseases, as detailed in Appendix A. Emergent themes are summarized in Appendix A, Table S2. Expectations and preferences for genomics to diagnose rare childhood diseases included improved management strategies, reduced stress and anxiety, increased knowledge, access to community supports, as well as contribution to scientific advancement. Perceived barriers and sources of concern included the inability to access sequencing and treatment, uncertain or inaccurate results, the return of an unfavorable prognosis, as well as the potential for misuse of personal and highly sensitive data. Following the development of the focus group guide, the research team provided feedback for clarity and length and to ensure the inclusion of pertinent topics. The guide included prompts to ensure relevant thematic areas were captured, as described in Table 1.

**Table 1:** Focus group topic guide.

Category	Focus group question
Introductions	What are your first thoughts about genomic testing to help learn more about why your child is ill?
Expectations and concerns	What are some benefits of genomic testing you would expect?
	What are some concerns you might have about genomic testing for your child?
Decision making and consent	What would be the most important thing to know before deciding whether or not to have your child undergo genomic testing?
	Who would you want to talk to or be involved in the decision to undergo genomic testing for your child?
	If the information obtained through your child’s clinical diagnostic test were to be made available for research purposes, would you be willing to consent? Why or why not?
Return of results	What kind of information would you want to receive from your child’s genomic test?
	How would you want to learn about the results of your child’s genomic test?
	What are your thoughts on sharing your child’s test results?

### Participant eligibility and recruitment

Eligible participants were English-speaking parents of unaffected children, age 19 years or older. Potential participants were screened for eligibility, diversity (age and sex) and excluded if they had a personal history with genetic testing to diagnose themselves or a close family member (such as their child) with a genetic condition. Individuals who had undergone routine prenatal testing or genetic testing for non-medical reasons (e.g., Ancestry.ca or 23andMe etc.) were eligible for participation.

Vancouver participants were recruited through a market research firm, Mustel Group, using a general population panel via random digit dial. Oxford parent participants were recruited through social media platforms including Twitter, Reddit, and Facebook. Individuals were invited to attend a single, face-to-face focus group lasting 90 minutes in duration. Using Mustel’s general population panel, investigators applied a purposive sampling technique with the intention of recruiting a balance of age and sex within each group. Analysis commenced immediately following the return of the first focus group’s transcripts. Recruitment for subsequent focus groups continued until two qualitative analysts (SP and JD) reached consensus on thematic saturation across all focus groups.

### Study process

Focus groups were conducted between January and February 2020. All focus groups were in-person and facilitated by one of two PhD researchers with experience conducting qualitative research (SP or DAR). The facilitator was joined by a note taker, as well as up to three additional research team member observers. Observers introduced themselves before the focus group discussion begun but did not intervene or participate in any way in the discussion. Facilitators were not previously known to participants.

At the start of each focus group, the facilitator introduced her- or himself, and their role on the research project. Following introductions, facilitators presented an educational slide deck to introduce key concepts (see Appendix B). The educational deck was designed to prime participants about concepts related to evidentiary and outcomes uncertainty associated with genomic testing in this clinical context. The presentation begun by defining the concept of rare diseases in terms of individual prevalence, population level burden, and phenotypic heterogeneity. Facilitators then discussed the process that patients and families endure while attempting to obtain a diagnosis. This was described as including multiple healthcare appointments, tests, as well as the potential for ineffective treatments. We then introduced genetic testing as a means to reduce the diagnostic odyssey and provide earlier, more effective disease management strategies, alongside uncertainty related to the potential for patient benefit. This discussion was framed around the possibility of not receiving a pathogenic finding, of receiving an actionable finding wherein effective treatments are either unavailable or inaccessible, as well as the potential for variants of unknown significance. The term “genetic” testing was used as facilitators discussed testing in context to “genetic disease.” Facilitators, during the presentation, defined terms related to both genetic and genomic testing. Finally, facilitators provided an introduction to the role of genetic counsellors as a resource to assist patients and their families in understanding the process of genomic testing and making decisions following the return of sequencing results.

Due to the fact that the objective of the focus was to elicit values, preferences and concerns most salient to participants, the educational slide deck did not include a description of current policies and procedures, for example, related to reimbursement of genomic testing, data storage, or data protections. At the end of the 10-min presentation, participants were offered the opportunity to ask clarifying questions to the facilitators.

After the completion of the first focus group, the research team decided to add an additional task at the end of the semi-structured discussion by asking participants directly to indicate the top factors that would impact their decision to have their child undergo genomic testing to diagnose a rare disease. This exercise was implemented for or focus groups 2, 3, and 4.

Immediately following each focus group, facilitators recorded field notes to detail key discussion points, major themes and to adjust the topic guide. Vancouver participants were mailed an honorarium of CAD$100.00 following the session and Oxford participants were provided with a gift card of their choice valued at £50. This study was approved by the BC Cancer Behavioral Research Ethics Board (REB H19–01087) and the University of Oxford Central University Research Ethics Committee (R56197/RE003). All participants provided written informed consent prior to each focus group. All signed forms were received and archived by the study coordinator.

### Analysis

Audio recordings were transcribed by a professional transcription company external to the research team, de-identified, and reviewed for accuracy prior to analysis. Using grounded theory to guide thematic analysis, data collection and analyses proceeded concurrently [40]. Three analysts (SP, JD, and FM) applied an iterative, constant comparative approach using QSR International’s NVivo 12 software [40–42]. Codebook development began with three analysts (SP, FM, and JD) independently reviewing large text selections to identify and discuss in vivo codes. Analysts refined the coding framework through regular meetings to discuss, break down, and merge coding categories. Once coding categories were agreed upon, two analysts (SP and JD) coded the remaining transcripts. Major themes were identified as an iterative process of categorizing codes by thematic construct. Member checking was not conducted.

Following the return of participant ranking exercises, one analyst (FM) categorized responses into broad themes, categorizing like responses and binning them into descriptive categories. Emergent themes were discussed with the research team to determine final categories. A single point was assigned for each response category. Results were tabulated and reported descriptively.

## Results

Four focus groups took place between January and February 2020. Owing to study logistics, the first three focus groups took place in Vancouver (focus groups 1–3), and the final session took place in Oxford (focus group 4). Following the analysis of the Oxford focus group, two analysts (SP and JD) reached a consensus that emergent themes were consistent with those having been identified in the Vancouver focus groups. For this reason, an agreement was reached that thematic saturation was achieved, further recruitment was no longer required. In total, 33 parents participated in this study. Focus groups lasted ≈90 minutes in duration. Table 2 describes participant characteristics.

**Table 2:** Self-reported participant demographics.

	CAN # (%)	UK # (%)
Gender		
Male	11 (47.8)	2 (20)
Female	12 (52.2)	8 (80)
Age		
18–34	5 (21.7)	3 (30)
35–49	8 (34.7)	3 (30)
50+	10 (43.5)	3 (30)
Missing/unclear	0 (0)	1 (10)
Ethnicity		
Indigenous (First Nations, Métis, Inuk /Inuit)	2 (8.7)	0 (0)
White	12 (52.2)	8 (80)
Black (e.g., Haitian, Afro-Canadian, Somali, Nigerian, Black British)	1 (4.3)	1 (10)
UK: Mixed Ethnicity	0 (0)	1 (10)
South Asian	1 (4.3)	0 (0)
East or South-East Asian	7 (30.4)	0 (0)
Education		
High school diploma or equivalent (UK: 2 or more A Levels)	2 (8.7)	1 (10)
Apprenticeship or trades certificate	1 (4.3)	0 (0)
College, CEGEP, or other non-university certificate or diploma	7 (30.4)	0 (0)
University certificate or degree at bachelor’s level or above	12 (52.2)	9 (90)
Other	1 (4.3)	0 (0)
Annual Household Income		
$25,000–$49,999 CAD (UK: £14,001–£30,000)	4 (17.4)	0 (0)
$50,000–$74,999 CAD (UK: £30,001–£44,000)	4 (17.4)	6 (60)
$75,000 and aboveCAD (UK: £44,001 and above)	15 (65.2)	3 (30)
Prefer not to answer	0	1 (10)

Fourteen codes were defined, and the codebook is presented in Table 3. Codes were not mutually exclusive, in that text selections could be coded within multiple coding categories.

**Table 3:** Codebook.

Decision making process	Persons involved and resources required to make decisions through testing and follow up period
Child’s involvement	Preferences for children’s involvement in the decision making, ownership, and the return of results
Preferences for sequencing and outcomes	Preferences related to the testing process and required outcomes
Severity of illness	Impact of disease severity on psychological distress, willingness to pay, and willingness to undergo testing
Diagnostic accuracy	Levels of comfort with diagnostic accuracy, validity and the potential for misdiagnosis
Evidentiary uncertainty	Uncertainty related to disease characterization, pathogenicity, and the likelihood of obtaining personal quailty of life and survival improvements following genomic testing
Parental psychological impact	Parental experienced and perceived stess and anxiety associated with having an ill child
Costs	Direct and indirect costs associated with testing. Includes both out-of-pocket and health system costs
Test access considerations	Expectations and concerns about access to testing (e.g., availability and cost)
Return of test results	Processes related to the return of test results, information returned, and the process of communicating results to family
Role for research	Opinions about developing an evidence base to support research
Data sharing	Cross-institutional and cross-jurisdictional data sharing (not specific to research purposes)
Data privacy and security	Concerns and requirements related to data privacy and security

### Emergent themes

To describe parental expectations, hopes, and concerns related to the use of genomic sequencing to diagnose rare childhood disease, the thematic analysis yielded four major themes, (1) variation in parental expectations of benefit, (2) improved management through research, (3) willingness to pay under conditions of uncertainty, and (4) managing uncertainty through process integration.

#### Variation in parental expectations of benefit

Discussions around how to characterize benefit were central across focus groups. The concept of “benefit” was defined both in terms of psychological benefit in the form of stress and anxiety alleviation, as well as in terms of clinical benefit in the form of improved health outcomes. Regarding the potential for psychological benefit, willingness to participate in genomic testing was often framed around the desire to alleviate stress associated with having an undiagnosed child. Participants prioritized interventions that could alleviate pre- and post-sequencing anxiety but expressed varying opinions in terms of how this could be accomplished. This is exemplified by participant’s interpretation of the value of a diagnosis in the absence of management options.

*Quote 1: “But I think as a parent just after a child’s been ill for months or years you’d just be desperate to know what’s going on when a visit to this doctor, to this doctor and that specialist and that specialist, nothing’s returned you that has helped you… I think you’d just get to the point where you’d forgo any other concerns, just like, “What is it? I need to know.”” [focus group (FG)1 participant(P)2]*

*Quote 2: “just that peace of mind saying I know what the problem is…” (FG4P11)*

*Quote 3: “… even if I didn’t get the result that was expected… that’s another level of weight off your mind…” (FG4P3)*

While some participants valued the indirect benefit of a diagnosis through the avoidance of travel and time away from work during the diagnostic odyssey, others acknowledged the ability to plan for their child’s care needs even in the absence of clinically actionable results. Within such discussions, the value of a diagnosis was––by some––weighed against the potential for diagnostic inaccuracy. While some participants acknowledged the value of no longer having to search for a diagnosis even in the absence of clinical utility, there was a consistent expectation that results would be accurate.

*Quote 4: “…just knowing what it is and knowing that there’s no course of treatment actually lets you develop a plan and figure out what that means and lets you have that certainty in your life that you probably need in a very uncertain time when your kid is sick. But if it’s a two per cent chance of that actually being correct and you have misdiagnosed it… so now you make certain decisions based on that, and you stop looking for other causes…that is very problematic in my mind.” (FG2P2)*

Although certain participants anticipated a sense of comfort by the resolution of the diagnostic odyssey, others argued that valued benefit would require access to effective disease management strategies. Here participants prioritized interventions with a high probability for clinical benefit.

*Quote 5: “There are no treatment options, I think would be a different type of stress.” (FG1P5)*

Participants frequently discussed obtaining a diagnosis in the absence of accessible treatment, some arguing that the inability to afford available treatments would prevent them from having their child sequenced.

*Quote 6: “… you wouldn’t want to know the results and know that it’s treatable if you couldn’t afford the treatment, because then you wouldn’t feel like a very good parent…Sorry, kid, I just can’t afford medical. At least we know.” (FG1P2)*

Participants prioritized genomic testing to guide decisions to improve familial and affected children’s wellbeing. We identified variation in terms of the extent to which participants would be willing to engage in genomic testing in the absence of clinical benefit, as well as the extent to which stress and anxiety would be resolved if no effective disease management strategies could be offered.

#### Improved management through research

A strongly voiced motivator for participating in genomic testing was to broaden the evidence base upon which a causal diagnosis could be more quickly and accurately established. Consistently, participants supported cross-jurisdictional data sharing and expressed concern over siloed data storage. Discussions yielded support for cross-jurisdictional efforts to identify patients with shared phenotypes.

*Quote 7: “…it’s not just about us in Canada whereas globally maybe there’s lots of people dealing with X in the U.K. and we can learn from that. It’s about sharing the data that we have.” (FG3P8)*

*Quote 8: “Other countries might have the disease and you don’t know so the more you know then the more resources could be pulled” (FG4P2)*

Alongside supporting a broader evidence base to improve diagnostics and management, participants discussed the need for large and cross-jurisdictional datasets. Within such discussions, parents acknowledged the sensitive nature of genomic data and the value of being made aware of how data security would be maintained, with whom it would be shared, and for what purposes.

*Quote 9: “Where is the information housed? Who has access to it? How is it identified? Those are all things that could concern me as a parent.” (FG3P2)*

Participants emphasized the importance of clear and transparent communication about the use of their child’s data for research purposes.

*Quote 10: “… make it crystal clear up front in really clean language that…“This is what we will be doing and how we’ll be using your genetic data for the research,” then at least you have the option to opt in.” (FG2P2)*

Support for data sharing and optimism for downstream benefit was considered alongside concerns about data access. As such, concerns centered around the potential for private industry to profit substantially through the development of high-cost treatments using donated genomic information. In particular, participants were uncomfortable with the development of prohibitively costly treatments.

*Quote 11: “You always hear about these medications that when they do get developed the cost is so astronomical that parents need to crowd source to pay for it because the provincial government doesn’t…”(FG1P2)*

Parents valued assurances that information would be maintained in a secure manner, contingent on explicit access permissions.

#### Willingness to pay for sequencing under conditions of uncertainty

A primary objective of our focus groups was to determine where participants would be willing to engage in sequencing under varying scenarios. One mechanism by which trade-offs can be elicited is by asking participants to consider hypothetical situations requiring out-of-pocket payment for testing. This hypothetical scenario reflects jurisdiction-specific current practice wherein families are required to pay-out of pocket to receive diagnostic sequencing. Explicit trade-offs were framed around the likelihood of personal health benefit, supporting the argument that while participants valued obtaining a diagnosis, willingness to pay was a direct function of likely clinical benefit.

*Quote 12: “… Am I going to spend all this money for – that isn’t even going to help my child at all, it’s just for research. Then I couldn’t, right?” (FG1P3)*

In some cases, participants reported being unconcerned with out-of-pocket payment given a high probability for personal benefit:

*Quote 13: … if you get the answer and it’s going to help your child or you deal with what’s going on, you will find the money if you can” (FG4P2)*

Participants were increasingly willing to pay for tests with a greater chance of offering enhanced treatment options for their own child, alongside diagnostic accuracy. Higher willingness to pay was voiced as a function of disease severity and expected clinical benefit. Fear of unnecessary or costly diagnostics, in addition to the potential for inappropriate treatment formed much of the discussions around out-of-pocket payment.

#### Managing uncertainty through process integration

Complex decision making under conditions of uncertainty were central to focus group discussions. Participants spoke at length about concerns related to the decision to undergo testing and how to move forward on the basis of sequencing results. In advance of decision making, participants discussed the provision of information about test procedure, invasiveness, cost, format, and presentation of forthcoming test results, how to interpret results carrying a range of certainty. Participants hoped to receive information in a digestible manner, with the support of their children’s healthcare providers. As a means to assist the decision-making process, participants were highly receptive to the role of supportive resources such as genetic counsellors.

*Quote 14: “…coming at it as someone with no great knowledge of how these things work, yeah, I would want some good briefing before going through this kind of thing.” (FG4P10)*

Some offered suggestion that genetic counsellors would be a valuable resource to facilitate complex decisions between parents and their children about their diagnosis, following the return of sequencing information.

*Quote 15: “Well, I think the genetic counselor would play a huge, huge, role… to help guide you so you can make decision, and you know, fact base and just how much do you tell your kid? How old are they?” (FG1P3)*

Acknowledging the wait time between sequencing and the return of test results, participants anticipated a need for ongoing support to manage anxiety and uncertainty. Further, participants anticipated the need to support families in managing their children’s health and wellbeing in both the short- and long-term, following the return of sequencing results. This included calls for the integration of individuals who could assist with both cognitive and physical development, as well as family supports.

*Quote 16: “… you might as a parent want to know the diagnosis when your child is fairly young, but then you’d also want the support to be able to… give them those age-appropriate messages as you go through… You need continuous support which is a lot to ask of the health service.” (FG4P6)*

Within these discussions, participants considered the potential downstream familial stress associated with the identification of germline variants, and how associated parental blame and guilt could potentially be managed using resources such as genetic counsellors.

### Decisional factors when considering genomic testing for rare childhood genetic disease

Table 4 illustrates the results of the decisional attribute ranking exercise. We report a list of decisional attributes that participants ranked as being first, second, and third most important features when making a decision about participating in genomic testing to diagnose children with a suspected rare genetic disease.

**Table 4:** Participant identification of the top three factors important for decisions to undergo genetic testing.

	Rank 1	Rank 2	Rank 3	Total
Privacy and ownership of data and results	4	6	4	14
Test accuracy/reliability	7	2	3	12
Test cost	1	6	4	11
Impact on clinical management	5	1	2	8
Reciept of causal diagnosis	2	1	1	4
Support services	1	1	2	4
Patient/parent choice or consent	1	2	1	4
Delivery of results (format)	0	1	2	3
Severity of illness/impact of illness on quality or length of life	2	0	0	2
Contribution to research evidence base	0	1	0	1
Other	1	3	3	7

Participants prioritized data privacy and ownership as one of their top three most important decisional factors (14/33, 42%), test accuracy (12/33, 36%) and cost considerations (11/33, 33%). Other decisional factors identified as important included clinical utility, the receipt of a causal genetic diagnosis, and decision support, including the format for the return of genomic test results. Findings illustrate the variation in participant values that persisted across focus groups. Participants considered a multitude of factors, both short- and long-term, that would influence willingness to engage in genomic testing. Combined with the qualitative results of focus group discussions, results support a patient-centered approach to decision-making under conditions of uncertainty, as well as a need to integrate a spectrum of potential outcomes into the evaluation of genomic testing.

## Discussion

This qualitative analysis reports on public values for the clinical implementation of genomic testing to diagnose rare childhood genetic disease under explicit conditions of uncertainty. Previous research has elicited and quantified value for genomics to diagnose rare childhood genetic disease from the perspective of patients and parents of affected patients [1, 20, 32, 43–49]. The generalizability of existing evidence is hampered by single centre analyses, disease- and test-specific values elicitation, as well as a lack of public values that explicitly integrate evidentiary and outcomes uncertainty into discussions. Importantly, existing studies have failed to integrate trade-offs for research and data sharing to support evidence development. Unique to our study is the characterization of parental perspectives for contributing patient data to research under conditions of uncertain clinical benefit. This work allows for a capture of the spectrum values underlying genomic testing to inform the development and administration of a multi-country discrete choice experiment. In particular, this work allows for more direct integration of uncertainty related to evidence and downstream outcomes than has previously been reported.

Our results illustrate parental support for genomic testing to generate evidence and enhance management strategies for children with a rare disease. Consistent with emerging policies and legislation to ensure data protection and the prevention of genetic discrimination [50], participants valued the development of an enhanced research evidence base alongside a secured approach to cross-jurisdictional data sharing. Parents prioritized an expectation of diagnostic accuracy and the integration of resources to assist complex decision making throughout the sequencing trajectory. In light of uncertain clinical benefit, participants varied considerably in terms of the extent to which they would be willing to engage with genomic testing, if testing were to incur out-of-pocket payment. Finally, this work highlights a need for ongoing supportive resources to assist families in making complex decisions, throughout the sequencing process and following the return of results.

The results presented here are consistent with existing evidence reporting values from parents of affected children [19, 47, 51]. Findings reflect previous qualitative work suggesting that a diagnosis alone is a necessary but insufficient condition to alleviate parental stress and anxiety [47], and that parents are primarily motivated by the potential for improved disease management for their children [19]. Our findings are consistent with previously reported support for genetic counselling throughout the testing and care trajectory [25, 47, 52], as well as guidelines recommending counselling for families considering sequencing [53]. Our participants further echoed existing concerns about data privacy and security [25, 51].

The perspectives of those who have undergone sequencing to diagnose a child with a genetic disease are likely to differ from those of parents for whom such decisions are hypothetical. For example, through semi-structured interviews with parents of undiagnosed children, Rosell and colleagues reported a comparatively stronger preference for a diagnosis, irrespective of clinical utility [19]. Parents reported being motivated to obtain a more accurate understanding of life expectancy, to inform reproductive decisions, and to connect with other families with the same disease. Interviews reported by Inglese et al support this latter finding, suggesting that social supports following a diagnosis may be more highly valued for parents with a lived experience of having a child with an undiagnosed disease [52]. Further, perspectives elicited from parents of children having received sequencing results further illustrate that the return of information unrelated to clinical utility––such as future reproductive planning––may bear on perceptions of benefit [47].

Patient and public values for genomic information are an important factor driving uptake and delivery of population-wide benefits of precision medicine [32]. Results reported here suggest that diagnostic yield alone is insufficient for economic evaluations of genomic testing in rare diseases. Rather, non-health outcomes such as contribution to the research evidence base may be highly valued under certain scenarios. While QALYs are recommended across jurisdictions as best practice for cost-effectiveness analyses, they are unable to account for non-health outcomes that patients and publics value [54]. Broadening base case analyses to include patient-reported outcomes capturing all valued endpoints is critical to support the acceptable implementation of precision medicine.

Our findings should be interpreted in light of study limitations. Firstly, while the Canadian focus groups captured a broader range of perspectives in terms of age, gender and ethnicity, the majority of participants across the four focus groups were highly educated and predominantly self-reported white ethnicity. A more diverse sample would have enhanced the ability to generalize the interpretation of results. Recruitment via social media for the Oxford focus group did not yield as many responses as anticipated. For this reason, diversity related to certain demographic factors was not met. Despite this, recruitment continued until the point at which thematic saturation was reached, as determined by two qualitative analysts (SP and JD).

Secondly, concepts related to genomic testing in context to rare disease are complex. Although facilitators endeavored to prime participants to these topics using the educational slide deck, participants may have felt unable to engage meaningfully in discussions due to the complexity of issues under discussion. The educational slide deck was not intended to make participants content experts, but rather to provide sufficient information to enable the expression of preferences alongside underlying rationale.

Thirdly, certain topics that have been shown to bear on willingness to participate in genomic testing in this context were not discussed in our focus groups. For example, discussions related to accessing community supports following a diagnosis, the impact of a diagnosis on decisions related to future pregnancies, and access to clinical trials following a diagnosis were not discussed in detail. Owing to the 90-min time frame allocated to each focus group, facilitators applied an approach wherein topics most salient to participants were discussed in greatest detail. As a result, the complete spectrum of *all* potentially pertinent decisional attributes may not have been prompted for discussion.

Finally, we present values and preferences of individuals facing hypothetical decisions that may not be representative of choices when faced with an actual decision. Although further investigation into the external validity of stated preference research is warranted, emerging evidence supports the claim that hypothetical preferences reflect real-world decision-making [31].

## Conclusion

A key obstacle to implementing genomic testing to diagnose rare childhood genetic disease involves limited empirical evidence on public values to direct management and improve patient outcomes. Sustainable integration of genomic testing into clinical practice will require efforts to both manage and mitigate uncertainty. Eliciting public values prior to implementation requires policies and practices to align with stakeholder values while ensuring that resources and infrastructure are available to support informed decision-making. This work will inform a multi-country economic evaluation of willingness to pay for genomic testing to diagnose rare childhood genetic disease, ensuring the comprehensive integration of public values. Evidence generated through this economic evaluation supports the timely and appropriate adoption of genomics to diagnose and treat children with rare diseases in clinical practice.

## Supplementary information

Appendix A (no revisions)

Appendix B (no revisions)
