# Supplementary material for: Toward the diagnosis of rare childhood genetic diseases: what do parents value most?
Source: Eur J Hum Genet. 2021 Apr 26;29(10):1491–501. doi: 10.1038/s41431-021-00882-1 (PMC8484431; doi:10.1038/s41431-021-00882-1)
Supplement: Supplementary file 1 — Appendix A (no revisions) [file 41431_2021_882_MOESM1_ESM.docx]

**Introduction**

This review was conducted to inform the creation of our focus group guide by exploring factors influencing stakeholder views and preferences for diagnostic testing of rare diseases. Through a scoping review, we identified emerging barriers and facilitators that influence participant’s decisions to initiate genomic testing.

**Methods**

We conducted a scoping review of peer-reviewed articles in Medline (PubMed), published between January 2005 and July 2019. We chose 2005 as this was the year when genomic testing using next generation sequencing (NGS) technologies was being implemented in research settings.(1) Our PubMed search included keywords and MeSH headings related to rare diseases, preference elicitation and genomics. Multiple searches were run to ensure that a broad spectrum of eligible articles would be captured. We included English language qualitative studies involving interviews and/or focus groups, and preference elicitation studies applying discrete choice experiments and/or contingent valuation techniques in the context of rare diseases. We applied the ISPOR Rare Disease framework, which defines a rare disease as affecting fewer than 50 in 100,000 people.(2)

**Article Selection**

The following inclusion criteria was used to ensure the contents and results are this review were as relevant as possible: qualitative studies and preference elicitation studies in the context of genetic/genomic testing and rare diseases.

**Data Abstraction and Narrative Synthesis**

Two researchers (FM, SG) independently extracted information on: publication details, study type, clinical context, technology, study population, outcomes, and factors identified as influencing preferences for genetic or genomic testing. Quality appraisal was limited to recording stated and perceived study limitations and assessing transparency of reporting. Owing to heterogeneity in methods and approaches, we stratified this abstraction according to study type. For qualitative studies, we recorded major barriers and facilitators reported when considering the decision to initiate genetic and genomic testing. For preference elicitation studies, we recorded study endpoints, attributes and levels.

To identify general themes emerging in the literature, we compared factors across studies and documented those appearing in more than one article and/or discussed as being significant to preference determination. We then grouped similar characteristics emerging across multiple studies. Groupings were categorized according to frequency and identified common themes.

**Results**

PubMed searches yielded 26 eligible studies included in our narrative synthesis, described in Table S1. The majority of studies were published between 2014 and 2016 (62%, n=16) and originated in Canada and/or the United States (62%, n=16). Thirty percent (n=8) considered whole exome or whole genome sequencing, while the remaining studies considered other or unspecified genetic technologies. To identify participant views and preferences, most studies (69%, n=18) employed qualitative methods, with 54% (n=14) conducting interviews and 15% (n=4) using focus groups. Preference elicitation methods were applied in 35% (n=9) of studies, with discrete choice experiments (DCEs) used in 31% (n=9) and contingent valuation in 4% (n=1). One study (4%) conducted a contingent valuation supplemented by semi-structured interviews to assess stakeholder preferences.(3) The most common study perspective focused on families of patients with rare diseases (62%, n=16), followed by patients (35%, n=9), healthcare professionals (19%, n=5), and parents from the general public (12%, n=3). Eight studies (31%) considered the perspectives of multiple stakeholder groups.

*Table 1: Summary characteristics for included studies*

| **Characteristics** | | **Studies: n (% of total)** | | |
| --- | --- | --- | --- | --- |
|  |  | *Preference Elicitation* | *Qualitative* | *Total* |
|  |  | *n = 9 (35%)* | *n = 17 (65%)* | *n = 26 (100%)* |
| **Publication Year** | |  | | |
|  | 2010 and earlier | 1 (4) | 0 (0) | 1 (4) |
|  | 2011 to 2013 | 1 (4) | 3 (12) | 4 (15) |
|  | 2014 to 2016 | 3 (12) | 13 (50) | 16 (62) |
|  | 2017 to 2019 | 4 (15) | 1 (4) | 5 (19) |
| **Country** | |  | | |
|  | United States | 3 (12) | 6 (23) | 9 (35) |
|  | Canada | 2 (8) | 2 (8) | 4 (15) |
|  | United States & Canada | 0 (0) | 3 (12) | 3 (12) |
|  | Netherlands | 1 (4) | 3 (12) | 4 (15) |
|  | Australia | 0 (0) | 3 (12) | 3 (12) |
|  | United Kingdom | 1 (4) | 0 (0) | 1 (4) |
|  | France | 1 (4) | 0 (0) | 1 (4) |
|  | Multi-country | 1 (4) | 0 (0) | 1 (4) |
| **Test Type** | |  | | |
|  | Whole genome sequencing | 1 (4) | 3 (12) | 4 (15) |
|  | Whole exome sequencing | 1 (4) | 3 (12) | 4 (15) |
|  | Other genomic tests | 3 (12) | 2 (8) | 5 (19) |
|  | Unspecified genetic test | 4 (15) | 6 (23) | 10 (38) |
|  | Unspecified newborn screening | 0 (0) | 3 (12) | 3 (12) |
| **Study Population*** | |  | | |
|  | General public: parents | 1 (4) | 2 (8) | 3 (12) |
|  | Families of affected patients | 4 (15) | 12 (46) | 16 (62) |
|  | Patients/Patient representatives | 3 (12) | 6 (23) | 9 (35) |
|  | Healthcare providers | 1 (4) | 4 (15) | 5 (19) |
|  | General Public | 3 (12) | 0 (0) | 3 (12) |
| **Clinical Context*** | |  | | |
|  | Developmental/Intellectual Disability | 2 (8) | 2(8) | 4(15) |
|  | Specific rare genetic disorders | 1(4) | 11(42) | 12 (46) |
|  | Unspecified, genetic disorders | 3(12) | 5(19) | 8(31) |
|  | Genetic cancers (rare and not rare) | 3(12) | 3(12) | 6(23) |
|  | Multiple diseases | 4(15)) | 9(35) | 13(50) |
| **Study Type*** | |  | | |
|  | Interview | 0 (0) | 14 (54) | 14 (54) |
|  | Focus Groups | 0 (0) | 4 (15) | 4 (15) |
|  | Discrete choice experiment | 8 (31) | 0 (0) | 8 (31) |
|  | Contingent valuation | 1 (4) | 0 (0) | 1 (4) |
|  | Online discussion groups | 0 (0) | 1 (4) | 1 (4) |
| *Categories are not mutually exclusive | | | | |

Of the 26 included studies, 11 (42%) considered preferences for genomic sequencing to diagnose pediatric patients, and 2 (8%) studies examined preferences for non-pediatric patients. The remaining studies focused on older genetic technologies, with 9 (35%) studies examining testing in pediatric patients and 4 (15%) considering non-pediatric patients.

Clinical context varied considerably. Half (n=13) of studies addressed one specific genetic disease, while the remainder considered multiple genetic disorders [e.g. idiopathic developmental disabilities (n=4, 15%), cancers with rare genetic variants such as Li-Fraumeni syndrome or familial adenomatous polyposis (n=6, 24%), early onset diabetes (n=2, 8%), lysosomal storage deficiencies (n=2, 8%), Friedreich Ataxia, Retinitis Pigmentosa, Spinal Muscular Atrophy, and Dubowitz Syndrome (n=1)]. Eight of the 26 studies (31%) did not label a genetic disorder. Instead, they described a suspected but unknown genetic condition or a variety of hypothetical genetic disorders varying in penetrance or prevalence. A summary of each included study including clinical context, methods and results, is described in Table S2.

**Qualitative Studies**

*Perceived Benefits*

Despite variability in clinical context, several common themes emerged. The most commonly identified benefits of testing are categorized as: *Clinical Utility, Psychological Benefits, Knowledge, Managing the Future, and Future Research and Societal Benefit.*

*Clinical Utility:* Clinical utility refers to any test result that is expected to affect the clinical management of the patient or their condition.(4) Perceived clinical utility was among the most commonly reported motivations to undergo genetic or genomic testing, appearing in 83% (n=15) of articles.

*Psychological Benefits:* Participants across studies frequently discussed psychological benefits as an expected or desired outcome of genetic testing (72%, n=13), which ranged from psychosocial effects, to control, to closure. Psychosocial effects included reduced anxiety, uncertainty, and guilt; a sense of relief at a diagnosis or ruling out of a condition; and improved social relations through accessing a community of other diagnosed individuals.

*Knowledge:* Participants in 72% (n=13) of studies expressed that acquiring knowledge through the test would be intrinsically valuable. “Knowledge is power” was a quote exemplifying this, appearing in Alderfer et al.’s(5) study of young adults with Li-Fraumeni Syndrome.

*Managing the Future:* In 83% (n=15) of studies, participants found value in genetic testing for its future utility. Participants expressed hope that future scientific advances could change the clinical actionability of their test results, through reduced uncertainty or emerging treatments options.

*Future Research and Societal Benefit:* Participants often stated that they were motivated to undergo genetic testing when their decision could benefit others (56%, n=10). Participants discussed the hope that their genetic information could contribute to a body of knowledge, or help future generations, even if test results did not provide personal benefit.

*Barriers*

Participants discussed a range of barriers to genetic testing, broadly categorized as:

*Testing Process, an Absence of Clinical Utility, Psychological Harms, Disutility of Knowledge, and Privacy and Ethical Issues.*

*Testing Process*: Participants in 61% (n=11) of studies listed process barriers as limiting uptake of genomic testing. Concerns included the potential for painful test procedures; wait times for the return of results; informational complexity; resistance from health care professionals; bureaucratic hurdles with accessing desired tests; and test cost.

*Absence of Clinical Utility:* In 61% (n=11) of studies, participants expressed concern that their genetic test results would not provide information that could guide healthcare decisions. Reported fears included the return of uncertain information that would prolong the diagnostic odyssey, inaccurate results, as well as the return of information with unknown clinical utility.

*Psychological Harms:* In 72% (n=13) of studies, participants expressed concern about negative psychological impacts that could result from genomic testing. Perceived harms included but were not limited to anxiety, hopelessness, or a “loss of childhood”; as well as implications for familial and romantic relationships.

*Disutility of Knowledge:* Participants in 50% (n=9) of studies discussed negative utility for the return of various forms of genomic information such as sequencing results that could have been actionable had they been tested earlier would be information that they would not want to receive.

*Privacy and Ethical Issues:* In 72% (n=13) of studies, participants expressed concerns about the potential for genomic testing to violate privacy and autonomy, particularly when patients were unable to provide informed consent, owing to age or developmental disability. Privacy concerns centered on the misuse of data, employer or insurer access to data, genetic discrimination, or later use of genetic data for purposes misaligned with patients’ values.

**Preference Elicitation Studies**

Preference elicitation studies varied according to study population, clinical context, model types, choices of attributes, and endpoints. Elements spanned five thematic areas consisting of: *Disease Characteristics, Clinical Utility, Privacy, Process Traits, and Type of Information.*

*Disease Characteristics:* Commonly described disease characteristics included penetrance and prevalence, as well as prognosis in the absence of treatment (e.g. potential disability, rate of progression, age of onset, and severity). This theme appeared in 44% (n=4) of preference elicitation studies.

*Clinical Utility:* Attributes pertaining to clinical utility considered effective treatment availability, actionability, diagnostic yield, and a diagnosis facilitating access to gated services. Seven (78%) of the preference elicitation studies considered attributes relating to clinical utility.

*Privacy:* Attributes involving privacy of testing were elicited in 22% (n=2) studies. For example, specific attributes included risk of genetic discrimination as well as the potential for shared access to test results.

*Process Traits:* Process attributes appeared in 78% (n=7) of preference elicitation studies. Examples include out of pocket cost of testing, the number of required tests, type of technology used, time to results, and test accuracy.

*Type of Information:* The potential for multiple streams of genomic information was considered in 44% (n=4) of the studies. Attributes included the identification of carrier status, variants of unknown significance, or secondary findings, as well as the extent to which a test could provide prognostic or diagnostic information.

**Translation of findings into focus group topic guide**

We integrated our findings into the focus group topic guide by designing questions that would ensure that identified expectations, barriers and facilitators could emerge naturally, if relevant to participants. Given our research objectives, we included additional interview prompts specifically related to information and supportive resources required to enable an informed decision to undergo testing, as described in table S1. We also asked about the conditions under which participants would be willing to consent to the use of their genomic information for the purposes of research. We finalized our focus group topic guide through consultation with all members of the research team, including clinicians and geneticists, and achieved consensus on included questions.

| **Table S1: Thematic analysis of structured literature review on genomic testing** | |
| --- | --- |
| Expectations and preferences for genomics to diagnose rare childhood genetic disease | Concerns and barriers to genomics for diagnosing rare childhood genetic disease |
| - Improved disease management - Psychological benefits for parents - Enhanced knowledge - Access to a community of diagnosed individuals - Potential for future scientific advancement | - Uncertain or inaccurate results - Confirmation of a diagnosis with a poor prognosis - Lack of availability or access to testing and corresponding treatments - Data privacy and the potential for misuse |

***Table S2: Detailed Study Characteristics***

| **Study (first author, year)** | **Context** | **Country** | **Technology** | **Perspective** | **Study Population** | **Method** | **Themes and/or Attributes** |
| --- | --- | --- | --- | --- | --- | --- | --- |
| Alderfer, MA (2017)(5) | Examining patient attitudes and expectations towards pediatric genetic testing for Li-Fraumeni Syndrome (LFS). | United States and Canada | Genetic (germline TP53 genetic test) | Patients | Child and adolescent LFS patients who had been offered genetic testing, or had a relative offered testing, before age 22 | Interviews (telephone) | Facilitators: Clinical Utility; Knowledge; Psychological Benefit; Future research and societal benefit  Barriers: Testing Process; Psychological Harms; Disutility of Knowledge; Absence of Clinical Utility; Ethics |
| Alderfer, MA (2014)(6) | Examining parent perspectives towards the costs and benefits of pediatric genetic testing for LFS. Includes both predictive and diagnostic testing motivations. | United States and Canada. | Genetic (germline TP53 genetic test) | Parents (of affected children) | Parents of at least one child (< age 22) who had been offered TP53 genetic testing for LFS. Includes those who tested positive, negative, and status unknown. | Interviews (telephone and in-person) | Facilitators: Clinical Utility; Knowledge; Psychological Benefit; Managing the Future; Future research and societal benefit  Barriers: Psychological Harms; Disutility of Knowledge; Ethics |
| Boeldt, DL (2016)(7) | Studying the perspectives of patients and parents towards the benefits and drawbacks of receiving whole genome sequencing (WGS) for undiagnosed diseases. Include neurological, gastrointestinal, and hematological rare diseases. | United States | WGS | Patients and Parents (of affected children) | Patients and parents of pediatric patients with undiagnosed rare diseases who had undergone genomic sequencing. | Interviews (telephone and in-person) | Facilitators: Clinical Utility; Knowledge; Psychological Benefit; Managing the Future; Future research and societal benefit  Barriers: Testing Process; Psychological Harms; Disutility of Knowledge; Absence of Clinical Utility |
| Bosma, AR (2015)(8) | Exploring patient experiences and views of genetic testing for maturity-onset diabetes of the young (MODY). | Netherlands | Genetic (type unspecified) | Patients and families | MODY patients, people with suspected MODY, and family (including parents) | Interviews (telephone and in-person) | Facilitators: Clinical Utility; Knowledge; Psychological Benefit; Managing the Future  Barriers: Disutility of Knowledge; Absence of Clinical Utility; Ethics |
| Eden, M (2013)(3) | Determining the willingness-to-pay (WTP) for genetic testing or a combination of genetic testing + counselling for Retinitis Pigmentosa (RP) | United Kingdom | Genetic test (type unspecified) + counselling, or genetic counselling alone | General public (disease-naïve), and disease-familiar | Disease-naïve: members of general public (University of Manchester employees)  Disease-familiar: members of RP rare disease advocacy group (patients and family) | Contingent Valuation (iterative bidding) + Interviews (in-person) | Facilitators: Clinical Utility; Knowledge; Psychological Benefit; Managing the Future; Future research and societal benefit |
| Hayeems, RZ (2016)(9) | Understanding parent opinions towards pediatric microarray, with goal of characterizing the concept of personal utility, and exploring how families understand aCGH results. | Canada | Microarray/array-based comparative genomic hybridization (CGH) | Parents (of affected children) | Parents of children who had received microarray analysis for autism, developmental delays, and congenital anomalies. | Interviews (telephone and in-person) | Facilitators: Clinical Utility; Psychological Benefit, Knowledge; Managing the Future |
| Joseph, G (2016)(10) | Examining parent opinions towards the use of WGS in expanded newborn screening for Primary Immunodeficiency Diseases (PIDDs). | United States | WGS | Parents (general public, and parents of affected children) | Two groups: pregnant women, and parents of children diagnosed with PIDDs | Focus Groups | Facilitators: Clinical Utility, Knowledge, Managing the Future  Barriers: Testing Process, Psychological Harms; Absence of Clinical Utility; Ethics |
| Kilambi, V (2014)(11) | Measuring personal utility of genetic testing for colorectal cancer (CRC) for people with at least one relative with a genetic diagnosis. | United States | Genetic test (type unspecified) | Public | General public, age > 50. Mix of disease-familiar and disease-naïve | Discrete Choice Experiment (DCE) | Disease Characteristics (disease risk); Privacy; Process Traits; Process Traits |
| Levenseller, BL (2013)(12) | Assessing stakeholder views on the incorporation of WES in clinical care, including preferences towards consent and return of results. Clinical context: mix of rare and non-rare diseases including bilateral sensorineural hearing loss, nuclear encoded mitochondrial respiratory chain disorders, sudden cardiac death, and autism spectrum disorders. | United States | Whole Exome Sequencing (WES) | Health Care Professionals (HCPs), Patients, and Families | Three stakeholder groups:  1. HCPs (bioethicists, genetic counsellors, physicians)  2. Parents of children with undiagnosed, suspected genetic diseases  3. Adolescent patients with suspected genetic disorders | Focus groups, and online discussion forum for adolescent patients | Facilitators: knowledge on condition, closure, value of knowledge, future planning, possibility of future use  Barriers: Testing Process; Psychological Harms; Disutility of Knowledge; Ethics |
| Lewis, MA (2018)(13) | Quantifying the effect of health condition characteristics on parental preferences towards genomic sequencing, for children with non-medically actionable genetic conditions. | United States | Genomic (genome-scale, type unspecified) | Parents (general public) | General public aged 18-40, parent of at least one child age <5, self-identifying as either White or Black. | DCE | Disease Characteristics; Clinical Utility |
| Li, KC (2016)(14) | Exploring parental perspectives towards decisional needs in considering genome-wide sequencing. | Canada | WGS | Parents (of affected children) | Parents of children with idiopathic and suspected genetic diseases, who had already consented to genomic sequencing. | Interviews (in-person) and Focus Groups | Barriers: Testing Process |
| Lisi, EC (2016)(15) | Eliciting the views of genetic HCPs towards the addition of 6 lysosomal storage diseases (LSDs) to neonatal screening, to inform policy. Diseases considered were MPS1, MPS2, Pompe, Gaucher, Fabry, and Krabbe: rare genetic diseases with mixture of early- and late-onset and varying treatability. | United States and Canada | Genetic testing (newborn screening, blood test) | HCPs | Genetic HCPs (including biochemical geneticists, genetic counsellors, medical geneticists), with experience working with metabolic/LSD patients | Interviews (telephone) | Facilitators: Clinical Utility; Psychological Benefit; Managing the Future  Barriers: Testing Process; Psychological Harms; Disutility of Knowledge; Absence of Clinical Utility; Ethics |
| Lowe, GC (2015)(16) | Examining the attitudes of patients and parents towards pre-symptomatic testing of children at risk for Friedrich’s Ataxia (FRDA), a rare disease with variable age of onset. | Australia | Genetic testing (type unspecified) | Patients and Parents (of affected children) | FRDA patients, and parents of children with FRDA | Interviews (telephone and in-person) | Facilitators: Clinical Utility, Psychological Benefit, Managing the Future  Barriers: Testing Process (HCP barriers); Psychological Harms (psychosocial negative); Absence of Clinical Utility; Ethics (childhood autonomy concerns) |
| Marshall, DA (2019)(17) | To determine parent valuation of clinical exome sequencing, estimating WTP and willingness to wait for changes in attributes, as well as a minimum acceptable chance of diagnosis for changes in each attribute. | Canada | Exome sequencing | Parents (of affected children) | Parents of children with rare and suspected genetic diseases | DCE | Clinical Utility (chance of diagnosis, medical benefit of diagnosis, diagnosis granting access to gated services); Privacy (risk of genetic discrimination); Process Traits (financial costs via WTP, time to results, testing technology); Type of Information (knowledge on the disease, information on family risk) |
| Meiser, B (2015)(18) | Determining the benefits and shortcomings patients perceive in relation to NGS, including their interest and information preferences. Clinical context: Hereditary cancer syndromes (including rare disease variants). | Australia | WGS and Panel testing | Patients | Adult patients with cancer diagnoses, and a >10% chance of carrying a pathogenic variant of a known cancer gene based off of family history. Patients must have undergone other types of genetic testing (germline mutation) in the previous 3 years with no genetic etiology delivered. | Interviews (telephone) | Facilitators: Clinical Utility (informing treatment), Managing the Future (future planning), Future research and societal benefit  Barriers: Testing Process (complex/overwhelming, financial costs); Psychological Harms (psychosocial negative); Disutility of Knowledge (preference for non-return of results); Ethics (childhood autonomy, data use concerns) |
| Peyron, C (2018)(19) | To determine parent preferences (and observe characteristics of preference heterogeneity) towards characteristics of WGS tests. | France | WGS | Parents (of affected children) | Parents of children with rare genetic diseases and developmental disorders. | DCE | Process Traits (financial costs to individual, support while awaiting results, Repeat analysis and validation), Type of Information (VUS and disclosure + decision-making, SF and disclosure + decision-making, ) |
| Regier, DA (2009)(20) | To determine parental WTP for different diagnostic technologies. | Canada | Array CGH and cytogenetic testing | Parents (of affected children) | Parents of children with idiopathic developmental disabilities (IDD) | DCE | Clinical Utility (proportion of patients receiving confirmed etiology); Process Traits (time to results, cost to individual) |
| Rosell, AM (2016)(21) | Exploring parental perceptions towards WES, including discussion of factors contributing to parental empowerment such as expectations, understanding the process and results, use of information, communication of findings. | United States | WES | Parents (of affected children) | Parents of children with undiagnosed disorders who had undergone WES. | Interviews (in-person and video call) | Facilitators: Clinical Utility (informing treatment), Psychological Benefit (positive psychosocial impacts, closure), Knowledge (value of knowledge, knowledge about the condition), Managing the Future (future planning), Future research and societal benefit  Barriers: Psychological Harms (distressing results); Disutility of Knowledge (preference for non-return/toxic knowledge); Absence of Clinical Utility (concerns on results returned); Ethics (data use) |
| Rothwell, E (2013)(22) | Studying attitudes of the general public towards newborn screening for rare genetic condition (spinal muscular atrophy), to study parental decision making in a pilot newborn screening trial. | United States | Genetic testing (newborn screening) | Parents (general public) | Members of the general public, aged 18+, with at least one child. | Focus Groups | Facilitators: Clinical Utility (informing treatment), Knowledge (value of knowledge), Future research and societal benefit  Barriers: Absence of Clinical Utility (concerns on results returned, prolonged diagnosis); Ethics (data use concerns) |
| Sapp, JC (2013)(23) | Characterizing parental preferences towards exome sequencing for their children and the various types of results that could be offered. | United States | Exome sequencing | Parents (of affected children) | Parents of pediatric patients participating in trio-based genomic sequencing. Proband diagnoses included: developmental delay, Overgrowth Syndrome, Dysmorphic features, Dubowitz Syndrome | Interviews (telephone) | Facilitators: Knowledge (knowledge on condition, value of knowledge); Clinical Utility (informing treatment), Psychological Benefit (control); Managing the Future (future planning, possibility of future use); Future research and societal benefit  Barriers: Psychological Harms (psychosocial negative); Absence of Clinical Utility; Ethics (childhood autonomy violation, data use concerns) |
| Severin, F (2015)(24) | Assessing the value judgments of various stakeholders in the prioritization of genetic tests, using hypothetical genetic diseases varying in severity and risk. | Multi-country (majority Europe) | Genetic test (type unspecified) | Multi-stakeholder: patients and professionals. Asked to act as a “healthcare decision-maker” | Stakeholder groups included patient representatives, members of the European Society of Human Genetics, and individuals registered on the EuroGentest project website | DCE | Disease Characteristics (disease risk, severity of condition); Process Traits (financial costs); Clinical Utility (medical benefit of diagnosis); Type of Information (aim of test) |
| van der Zwaag (2015)(25) | Determining the views of HCPs towards factors both positively and negatively affecting the uptake of genetic testing for the rare disease MODY. | Netherlands | Genetic test (type unspecified) | HCPs | HCPs with existing or potential future experience requesting genetic testing for MODY. Includes pediatric endocrinologist, clinical geneticist, etc. | Interviews (telephone and in-person) | Facilitators: Clinical Utility (informing treatment, accessing services); Knowledge (value of knowledge); Managing the Future (future planning); Future research and societal benefit  Barriers: Testing Process (HCP barriers, financial costs); Psychological Harms (psychosocial negative, distressing results); Absence of Clinical Utility; Ethics (data use concerns) |
| van El, CG (2014)(26) | Assessing the attitudes of HCPs towards the addition of Pompe diseases (rare genetic LSD) to regular neonatal testing, to inform policy-making in expanding screening to broad-phenotype disorders with the advent of new developments in enzyme replacement therapy. | Netherlands | Genetic testing (newborn screening) | HCPs | Experts in Pompe disease and HCPs involved in healthcare policy relating to neonatal screening. Included pediatricians, neurologists, clinical geneticist, midwife, physicians, members of neonatal screening organization | Interviews (in-person) | Facilitators: Clinical Utility (informing treatment); Knowledge (knowledge on condition, value of knowledge); Psychological Benefit (closure); Managing the Future (future planning); Future research and societal benefit  Barriers: Testing Process (complex/ overwhelming information, financial costs); Psychological Harms (psychological negative); Absence of Clinical Utility (concerns on results returned); Ethics (data use concerns) |
| Vears, DF (2016)(27) | Examining parental views towards genetic testing for unaffected siblings of children with genetic conditions. | Australia | Genetic testing (type unspecified) | Parents (of affected children) | Parents of children with one of 3 genetic conditions (Cystic fibrosis, hemophilia, Duchenne muscular dystrophy) with at least one other child, who have expressed interest in genetic testing for their unaffected (pre-symptomatic and asymptomatic) children. | Interviews (telephone) | Facilitators: Psychological Benefit (positive psychosocial impacts); Knowledge (value of knowledge); Managing the Future (future planning)  Barriers: Testing Process (HCP barriers); Psychological Harms (psychosocial negative) |
| Veldwijk, J (2016)(28) | To identify preferences for genetic testing for CRC (Lynch, familial adenomatous polyposis, familial colorectal cancer) for people with familial or personal cancer history. | Netherlands | Genetic testing (type unspecified) | Public, asked to consider testing for people with familial or personal cancer history | Members of general population aged 55-65 who had not undergone CRC screening | DCE | Disease Characteristics (disease risk; prognosis in absence of treatment); Clinical Utility (medical benefit of diagnosis); Process Traits (frequency of testing); |
| Weymann, D (2018)(29) | Enumerate patient preference-based utility and WTP for massively parallel sequencing for CRC risk | United States | Massively parallel sequencing (MPS) | Patients and people at risk of CRC | People with personal or familial history of CRC, polyposis, or other Lynch syndrome features who have not undergone genetic testing, or patients with suspected hereditary cancer. | DCE | Clinical Utility (proportion of patients receiving definite diagnosis); Process Traits (financial cost; number of tests, time to results) |

1. Regier DA, Weymann D, Buchanan J, Marshall DA, Wordsworth S. Valuation of health and nonhealth outcomes from next-generation sequencing: approaches, challenges, and solutions. Value in Health. 2018;21(9):1043-7.

2. Richter T, Nestler-Parr S, Babela R, Khan ZM, Tesoro T, Molsen E, et al. Rare Disease Terminology and Definitions-A Systematic Global Review: Report of the ISPOR Rare Disease Special Interest Group. Value in health : the journal of the International Society for Pharmacoeconomics and Outcomes Research. 2015;18(6):906-14.

3. Eden M, Payne K, Combs RM, Hall G, McAllister M, Black GC. Valuing the benefits of genetic testing for retinitis pigmentosa: a pilot application of the contingent valuation method. The British journal of ophthalmology. 2013;97(8):1051-6.

4. Pollard S, Sun S, Regier DA. Balancing uncertainty with patient autonomy in precision medicine. Nature Reviews Genetics. 2019;20(5):251-2.

5. Alderfer MA, Lindell RB, Viadro CI, Zelley K, Valdez J, Mandrell B, et al. Should Genetic Testing be Offered for Children? The Perspectives of Adolescents and Emerging Adults in Families with Li-Fraumeni Syndrome. Journal of genetic counseling. 2017;26(5):1106-15.

6. Alderfer MA, Zelley K, Lindell RB, Novokmet A, Mai PL, Garber JE, et al. Parent decision-making around the genetic testing of children for germline TP53 mutations. Cancer. 2014;121(2):286-93.

7. Boeldt DL, Cheung C, Ariniello L, Darst BF, Topol S, Schork NJ, et al. Patient perspectives on whole-genome sequencing for undiagnosed diseases. Personalized medicine. 2016;14(1):17-25.

8. Bosma AR, Rigter T, Weinreich SS, Cornel MC, Henneman L. A genetic diagnosis of maturity-onset diabetes of the young (MODY): experiences of patients and family members. Diabetic medicine : a journal of the British Diabetic Association. 2015;32(10):1385-92.

9. Hayeems RZ, Babul-Hirji R, Hoang N, Weksberg R, Shuman C. Parents' Experience with Pediatric Microarray: Transferrable Lessons in the Era of Genomic Counseling. Journal of genetic counseling. 2016;25(2):298-304.

10. Joseph G, Chen F, Harris-Wai J, Puck JM, Young C, Koenig BA. Parental Views on Expanded Newborn Screening Using Whole-Genome Sequencing. Pediatrics. 2016;137 Suppl 1:S36-46.

11. Kilambi V, Johnson FR, Gonzalez JM, Mohamed AF. Valuations of genetic test information for treatable conditions: the case of colorectal cancer screening. Value in health : the journal of the International Society for Pharmacoeconomics and Outcomes Research. 2014;17(8):838-45.

12. Levenseller BL, Soucier DJ, Miller VA, Harris D, Conway L, Bernhardt BA. Stakeholders' opinions on the implementation of pediatric whole exome sequencing: implications for informed consent. Journal of genetic counseling. 2013;23(4):552-65.

13. Lewis MA, Stine A, Paquin RS, Mansfield C, Wood D, Rini C, et al. Parental preferences toward genomic sequencing for non-medically actionable conditions in children: a discrete-choice experiment. Genetics in medicine : official journal of the American College of Medical Genetics. 2018;20(2):181-9.

14. Li KC, Birch PH, Garrett BM, MacPhee M, Adam S, Friedman JM. Parents' Perspectives on Supporting Their Decision Making in Genome-Wide Sequencing. Journal of nursing scholarship : an official publication of Sigma Theta Tau International Honor Society of Nursing. 2016;48(3):265-75.

15. Lisi EC, McCandless SE. Newborn Screening for Lysosomal Storage Disorders: Views of Genetic Healthcare Providers. Journal of genetic counseling. 2016;25(2):373-84.

16. Lowe GC, Corben LA, Duncan RE, Yoon G, Delatycki MB. "Both Sides of the Wheelchair": The Views of Individuals with, and Parents of Individuals with Friedreich Ataxia Regarding Pre-symptomatic Testing of Minors. Journal of genetic counseling. 2015;24(5):732-43.

17. Marshall DA, MacDonald KV, Heidenreich S, Hartley T, Bernier FP, Gillespie MK, et al. The value of diagnostic testing for parents of children with rare genetic diseases. Genetics in medicine : official journal of the American College of Medical Genetics. 2019.

18. Meiser B, Storey B, Quinn V, Rahman B, Andrews L. Acceptability of, and Information Needs Regarding, Next-Generation Sequencing in People Tested for Hereditary Cancer: A Qualitative Study. Journal of genetic counseling. 2015;25(2):218-27.

19. Peyron C, Pelissier A, Bejean S. Preference heterogeneity with respect to whole genome sequencing. A discrete choice experiment among parents of children with rare genetic diseases. Social science & medicine (1982). 2018;214:125-32.

20. Regier DA, Friedman JM, Makela N, Ryan M, Marra CA. Valuing the benefit of diagnostic testing for genetic causes of idiopathic developmental disability: willingness to pay from families of affected children. Clinical genetics. 2009;75(6):514-21.

21. Rosell AM, Pena LD, Schoch K, Spillmann R, Sullivan J, Hooper SR, et al. Not the End of the Odyssey: Parental Perceptions of Whole Exome Sequencing (WES) in Pediatric Undiagnosed Disorders. Journal of genetic counseling. 2016;25(5):1019-31.

22. Rothwell E, Anderson RA, Swoboda KJ, Stark L, Botkin JR. Public attitudes regarding a pilot study of newborn screening for spinal muscular atrophy. American journal of medical genetics Part A. 2013;161A(4):679-86.

23. Sapp JC, Dong D, Stark C, Ivey LE, Hooker G, Biesecker LG, et al. Parental attitudes, values, and beliefs toward the return of results from exome sequencing in children. Clinical genetics. 2013;85(2):120-6.

24. Severin F, Hess W, Schmidtke J, Muhlbacher A, Rogowski W. Value judgments for priority setting criteria in genetic testing: a discrete choice experiment. Health policy (Amsterdam, Netherlands). 2015;119(2):164-73.

25. van der Zwaag AM, Weinreich SS, Bosma AR, Rigter T, Losekoot M, Henneman L, et al. Current and best practices of genetic testing for maturity onset diabetes of the young: views of professional experts. Public health genomics. 2015;18(1):52-9.

26. van El CG, Rigter T, Reuser AJ, van der Ploeg AT, Weinreich SS, Cornel MC. Newborn screening for pompe disease? a qualitative study exploring professional views. BMC pediatrics. 2014;14:203.

27. Vears DF, Delany C, Massie J, Gillam L. Parents' experiences with requesting carrier testing for their unaffected children. Genetics in medicine : official journal of the American College of Medical Genetics. 2016;18(12):1199-205.

28. Veldwijk J, Lambooij MS, Kallenberg FG, van Kranen HJ, Bredenoord AL, Dekker E, et al. Preferences for genetic testing for colorectal cancer within a population-based screening program: a discrete choice experiment. European journal of human genetics : EJHG. 2016;24(3):361-6.

29. Weymann D, Veenstra DL, Jarvik GP, Regier DA. Patient preferences for massively parallel sequencing genetic testing of colorectal cancer risk: a discrete choice experiment. European journal of human genetics : EJHG. 2018;26(9):1257-65.
