# Supplementary material for: Toward the diagnosis of rare childhood genetic diseases: what do parents value most?
Source: Eur J Hum Genet. 2021 Apr 26;29(10):1491–501. doi: 10.1038/s41431-021-00882-1 (PMC8484431; doi:10.1038/s41431-021-00882-1)
Supplement: Supplementary file 2 — Appendix B (no revisions) [file 41431_2021_882_MOESM2_ESM.pdf]

BC CANCER
Provincial Health Services Authority

ARCC
Canadian Centre for Applied Research in Cancer Control

Your views on genetic testing for children with rare diseases

Focus Group Session

Housekeeping

- Your participation is voluntary
- There are no right or wrong answers
- Please allow everyone the chance to speak
- We ask for you to respect the privacy of your fellow focus group members

Why we have invited you here today

What will we be discussing?

Your opinions about genetic testing to diagnose rare diseases in children

How will we use our findings?

To shape health policy related to genetic testing for children with suspected rare diseases

Rare diseases:
Individually rare but affecting many

6,000 to 8,000 known rare diseases

Each affecting less than 1 in 2,000 people

1 in 12 Canadians are affected

Most rare diseases are genetic in origin

What is the diagnostic odyssey?

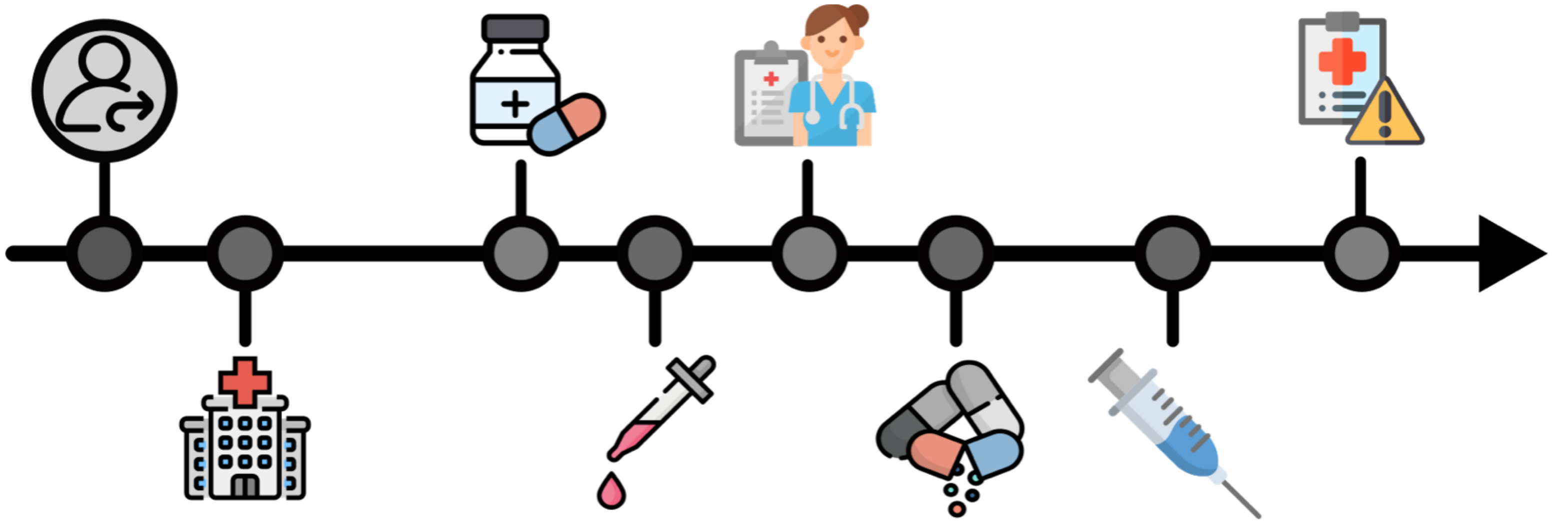

What is genetic testing?

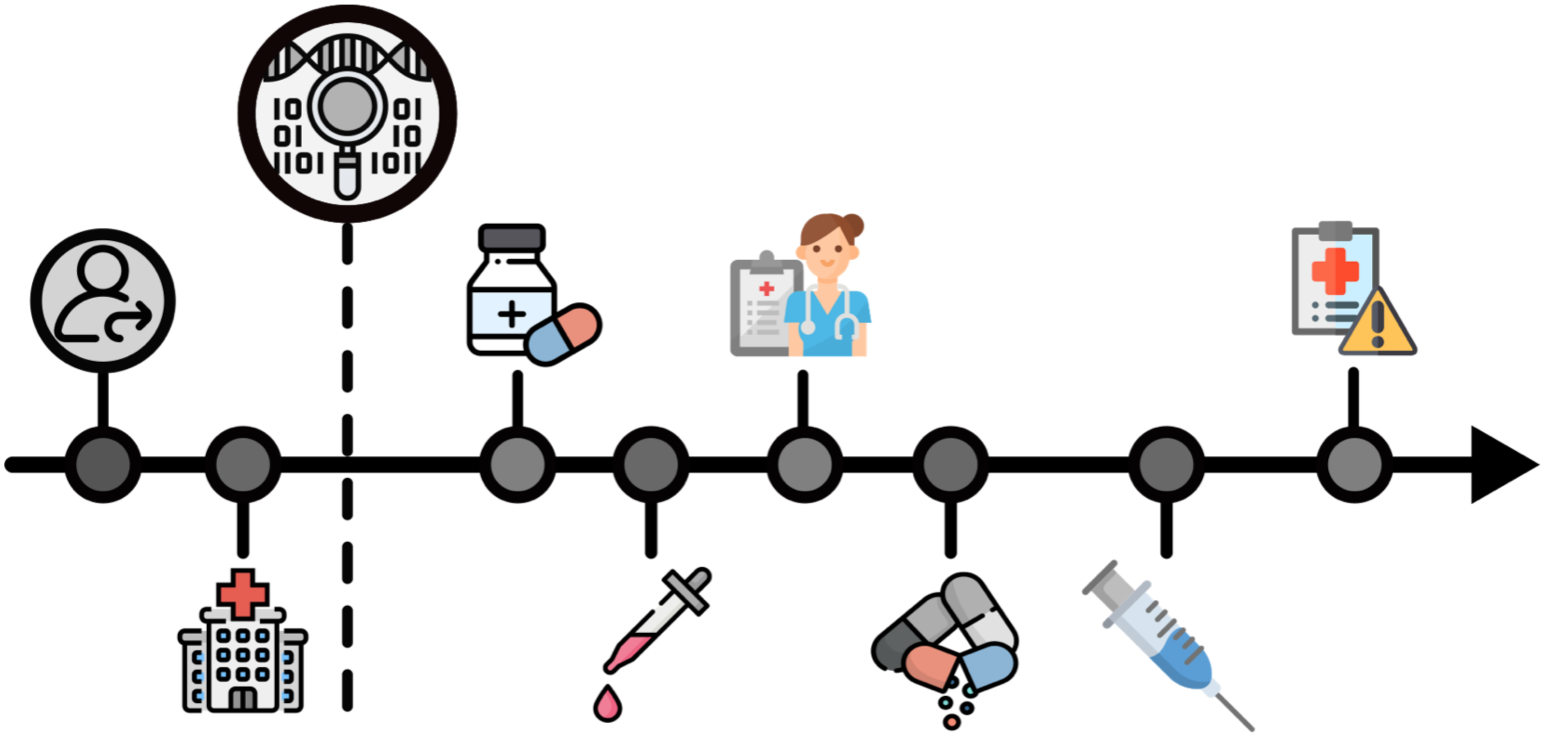

Test results

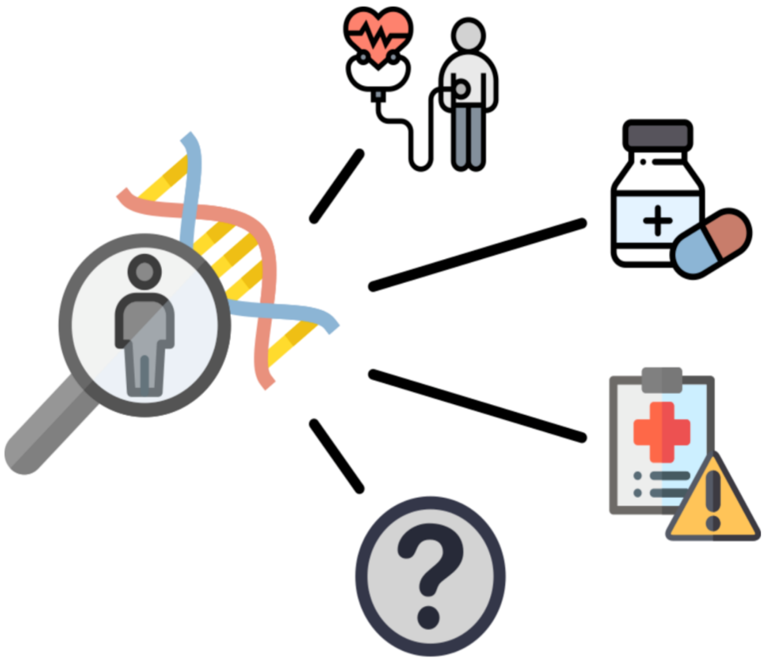

Earlier diagnosis

Better treatment

Disease risk

No information

Uncertain information

Supporting decision making

- Genetic Counsellors
  - Experts in genetics and genetic diseases
  - Provide education
  - Ensure your understanding
  - Discuss test results and next steps

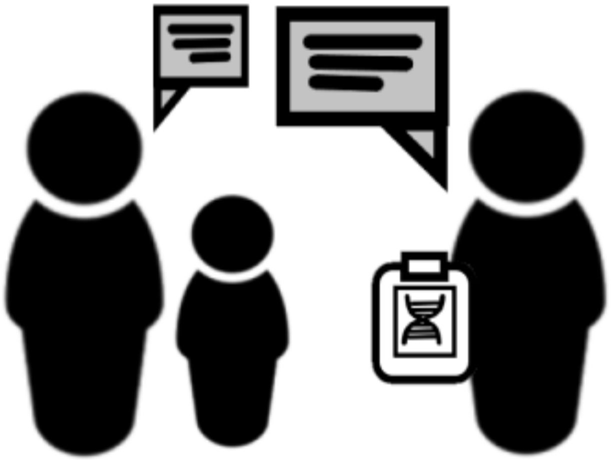

Today's discussion

Imagine that your child has a disability or severe illness. Doctors have not been able to find a cause.

What is important to you when making a decision about genetic testing for your child?
